# Social Recovery Therapy in improving activity and social outcomes in early psychosis: Current evidence and longer term outcomes

**DOI:** 10.1016/j.schres.2017.10.006

**Published:** 2019-01

**Authors:** David Fowler, Jo Hodgekins, Paul French

**Affiliations:** a University of Sussex, Brighton BN1 9RH, UK; b Norwich Medical School, University of East Anglia, Norwich NR4 7TJ, UK; c University of Manchester, Oxford Road, Manchester M13 9PL, UK

**Keywords:** Social recovery, Psychosis, Cognitive behaviour therapy

## Abstract

**Background:**

Social Recovery Therapy (SRT) is a cognitive behavioural therapy which targets young people with early psychosis who have complex problems associated with severe social disability. This paper provides a narrative overview of current evidence for SRT and reports new data on a 2 year follow-up of participants recruited into the Improving Social Recovery in Early Psychosis (ISREP) trial.

**Method:**

In the ISREP study 50 participants (86%) were followed up at 2 years, 15 months post treatment. The primary outcome was engagement in paid work, assessed using the Time Use Survey. Engagement in education and voluntary work were also assessed. In addition, the Positive and Negative Syndrome Scales (PANSS) and the Beck Hopelessness Scale (BHS) were administered.

**Results:**

25% of individuals with non-affective psychosis in the treatment group had engaged in paid work at some point in the year following the end of therapy, compared with none of the control group. Data from the PANSS and BHS suggested no worsening of symptoms and an indication that gains in hope were maintained over the 15 month period following the end of therapy.

**Conclusion:**

Social Recovery Therapy is a promising psychological intervention which may improve social recovery in individuals with early psychosis. The new data reported in this paper shows evidence of gains in engagement in paid employment outcomes that persisted 15 months beyond the period of active intervention.

## 1 Introduction

### 1.1 Background

Psychosis is the illness of working age adults most frequently associated with poor outcomes. A review of recovery rates suggests that, despite recent advances in treatment options, < 14% of individuals diagnosed with schizophrenia achieve sustained recovery on both symptomatic and functional outcomes (Jaaskeelainen et al., 2013). Social and functional outcomes from psychosis have received more attention in recent years and feature in service user definitions of recovery (Law and Morrison, 2014). Social recovery can be defined in terms of engagement in activities within occupational and interpersonal domains (Hodgekins et al., 2015a, Hodgekins et al., 2015b). This may include work, education, valued social activities, and relationships with others. Studies suggest that < 50% of people with non-affective psychosis achieve a social recovery (Hafner and an der Heiden, 1999, Harrison et al., 1996), and only 10–20% of people return to competitive employment despite the majority suggesting that they wish to work (Mueser et al., 2001). The personal and economic costs of this disability are large (Fleischhacker et al., 2014). The lives of young people are disrupted at a crucial stage of development and many continue to struggle over the long term to achieve key milestones in terms of personal achievement and social roles (Bond et al., 2014, Kam et al., 2013, Lenior et al., 2001, Wiersma et al., 2000).

### 1.2 Treating social disability in psychosis

Perhaps unsurprisingly due to their focus on positive psychotic symptoms, pharmacological treatments for psychosis appear to have no direct effects on functional recovery (Kern et al., 2009). Indeed, side effects from medication may even hamper activity levels. Early Intervention Services have demonstrated some success in improving social outcomes in first episode psychosis by providing assertive case management and supported employment interventions (Fowler et al., 2009a, Craig et al., 2014). However, despite provision of such services, a substantive proportion of cases remain socially disabled (Hodgekins et al., 2015a). More specific targeting of those individuals showing early signs of delayed social recovery in first episode psychosis using cognitive behaviour therapy (CBT) may be an important way to further improve the effectiveness of Early Intervention Services (Fowler et al., 2010).

A major success of CBT has been on targeted interventions which focus primarily on unitary disorders and single symptoms. Research trials of CBT for psychosis have shown promising indications of an impact on social disability where assessed as a secondary outcome. The systematic review of studies of CBT in psychosis carried out by Wykes et al. (2008) highlights an effect of CBT on social disability where assessed as a secondary outcome with a mean effect of 0.38 (15 studies), although social disability was not specifically targeted. The NICE (2014) schizophrenia review also reports an effect of CBT for psychosis on social functioning.

However, the challenge often faced in complex cases is comorbidity. Young people with first episode psychosis who do not recover socially often leave work or education and lose contact with social networks (Killackey et al., 2009, Bond et al., 2015, Kam et al., 2013). Such individuals often adopt lifestyle patterns of extreme social withdrawal, which typically occurs in the context of complex comorbid symptoms of paranoia and other positive and negative psychotic symptoms and frequently also depression, anxiety and other disorders (Hodgekins et al., 2015a). Alongside such issues are complex social circumstances and systemic issues including problematic family dynamics, victimisation, social threat and social deprivation. The cases at highest risk are the most complex, and a single symptom focused approach is not sufficient. Clinically, the presentations are complex and therapists can easily become overwhelmed and hopeless, not knowing where to start.

### 1.3 Social recovery therapy

We have developed a novel CBT intervention called Social Recovery Therapy (SRT; Fowler et al., 2013). The focus of the intervention is on the individual's values and goals, identifying problems and barriers to these, then promoting hope for meaningful behavioural change. Our approach is to start with a formulation of social recovery from the perspective of the individual. This provides a clear direction for both therapists and clients faced with what can seem otherwise an overwhelming clinical scenario. Cognitive techniques are used to develop a sense of optimism and agency and to build positive beliefs about self and others. There is a large emphasis on the use of behavioural strategies (including behavioural experiments, graded exposure and behavioural activation) to overcome avoidance and promote meaningful behavioural change “in vivo” whilst managing symptoms as necessary to address a meaningful pathway to social recovery. Evidence and experiences from this behavioural work are used to further instil hope and promote positive beliefs about self as the individual works towards achieving meaningful change in their lives.

SRT differs from traditional CBT for psychosis in its largely behavioural focus and emphasis on building positive beliefs about self and others rather than challenging negative beliefs in isolation. In addition, to achieve gains in social recovery against a background of often years of withdrawal and social disadvantage means that therapists have to integrate techniques more typically associated with assertive community treatment and supported employment. Working systemically with families and stakeholders surrounding the individual to promote opportunities in the social environment is also important.

### 1.4 Research evidence in support of SRT

To date, we have conducted two single-blind randomised controlled trials of SRT with individuals with first episode psychosis and social recovery difficulties: the Improving Social Recovery in Early Psychosis (ISREP) trial (Fowler et al., 2009b) and the Sustaining Positive Engagement and Recovery (SUPEREDEN) trial (Fowler et al., in press). In both studies, the primary outcome was hours per week spent in structured activity, assessed using the Time Use Survey (Hodgekins et al., 2015b).

In the ISREP trial, 77 participants with affective or non-affective psychosis were randomised to receive either SRT plus Treatment as Usual (SRT + TAU) or TAU alone. TAU consisted of case management from a secondary mental health care team. We found differential effects for people with affective and non-affective psychosis. Specifically, in the non-affective psychosis group, SRT showed significant superiority on the primary outcome of weekly hours in structured activity. In addition, significant superiority of SRT + TAU over TAU alone was seen for Positive and Negative Syndrome Scale (PANSS; Kay et al., 1987) scores. There was an effect of therapy on hopelessness and positive beliefs about self and improvements on these variables were a mediator of change in the therapy group (Hodgekins and Fowler, 2010). The intervention was also shown to be cost-effective (Barton et al., 2009).

The SUPEREDEN3 trial was a larger (N = 154) and more definitive multicentre trial of SRT conducted as part of a programme of research evaluating UK Early Intervention Services (Birchwood et al., 2014). SUPEREDEN3 tested the efficacy of enhancing social recovery following first episode psychosis by combining the use of standard Early Intervention Service (EIS) provision with Social Recovery Therapy (SRT). The primary hypothesis was that SRT in combination with EIS would lead to improvements in social recovery compared with EIS alone. Participants were also followed up 6 months after the end of the intervention.

The primary analysis indicated that the SRT + EIS was associated with an average increase in structured activity of just over 8 h per week greater than EIS alone (95% CI 2.5 to 13.6; p = 0.005). A consensus group of clinicians and service users have conservatively estimated the minimum clinically significant gain on the TUS as 4 h. The size of the effect in the SUPEREDEN3 trial is twice this gain and represents an amount of activity equivalent to a working day. As such, the findings show a clinically important benefit of enhanced social recovery for the SRT plus EIS group on the primary outcome of structured activity post-therapy. Modelling of outcomes 6 months after the end of the intervention also showed promise for the maintenance of therapy gains and improvements in trait hope.

### 1.5 Long-term outcomes and therapy gains maintenance

Both the ISREP and SUPEREDEN3 trials provide some evidence in support of SRT in producing clinically significant gains in time spent in structured activity compared to treatment as usual. There is also a suggestion that this gain may be maintained 6 months beyond active treatment. However, despite the development of new treatments, studies have found that long-term functional outcomes following psychosis remain poor (Jaaskeelainen et al., 2013). Therefore, evidence of longer term outcomes following SRT is required.

In addition to studying maintenance effects of SRT, a longer follow-up period would enable further changes in social recovery to be examined. A common goal of individuals taking part in the ISREP and SUPEREDEN3 trials was to return to work and education. Participants had often been unemployed for long periods of time prior to being recruited into the study and thus whilst weekly hours in structured activity improved following the delivery of SRT, it was anticipated that the full effects of the intervention on engagement in paid work may not be observed immediately post-intervention. Following the end of the intervention period it was often noted that participants were in the process of applying for work or educational programmes but that formal engagement in these activities had not yet commenced. A longer term follow-up would enable an investigation of whether work and education were taken up following the end of the intervention.

### 1.6 Aims and hypotheses of the current study

The current study reports on longer term follow-up data from participants who took part in the ISREP trial. Participants were followed up 15 months after the end of the intervention period (2 years following entry into the study) to explore whether or not they had engaged in work, education or voluntary work following the end of therapy. It was hypothesised that a greater proportion of the SRT + TAU group would have engaged in work, education or voluntary work when compared to the group who received TAU alone. Long-term effects of the intervention on symptoms and hopelessness were also examined as these variables were found to mediate outcome in the primary post-intervention analyses. Differences in outcomes for individuals with affective and non-affective psychosis were explored as the intervention showed differential effects for these groups post-intervention, with therapy effects being shown for the non-affective group only.

## 2 Method

### 2.1 Design

The ISREP trial was a single blind randomised controlled treatment trial comparing SRT in addition to treatment as usual (SRT + TAU) with those receiving TAU alone. All participants were receiving care from secondary mental health services and thus TAU involved regular contacts with mental health professionals, including Case Managers and Psychiatrists. However, participants in the control arm of the study did not receive any psychological therapy. See Fowler et al. (2009b) for a full description of the trial. In the current study, trial participants were followed up 2 years after randomisation had taken place, 15 months after the end of the intervention period.

### 2.2 Participants

**Fig. 1:**
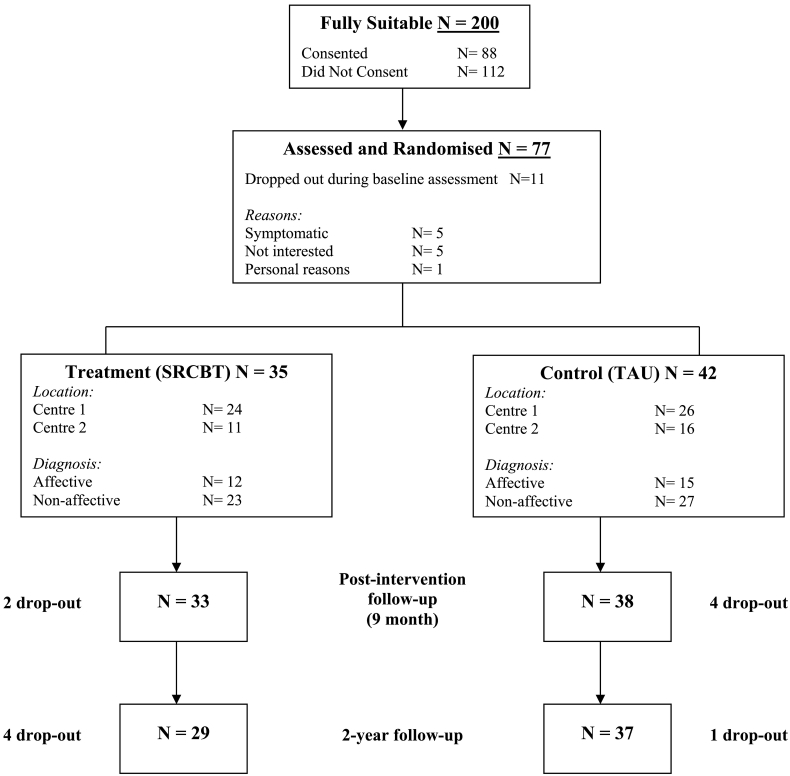
CONSORT diagram of flow of participants through the trial.

Inclusion and exclusion criteria and participant characteristics for the ISREP trial have been described in the trial outcome paper (Fowler et al., 2009b). Seventy-seven participants were originally recruited into the ISREP study: 35 were randomised to receive SRCBT and 42 were randomised to receive TAU. Of these, 66 (86%) were followed-up 2 years later: 29 (82.8%) of the SRCBT group and 37 (88%) of the TAU group. Of those 11 individuals who were not followed up at 2 years, 6 had dropped out of the study during the intervention period; 2 could not be contacted, and 3 declined to participate in the follow-up assessment (Fig. 1).

### 2.3 Measures

#### 2.3.1 Primary outcome

The presence of paid work, education, and voluntary work occurring at any point in the year following the end of therapy was screened for using the Time Use Survey (Hodgekins et al., 2015b, Gershuny, 2011). The TUS is a semi-structured interview assessing how individuals spend their time. Following the interview, work, education, and voluntary work were coded as being either present or absent in the year following the end of the intervention period. This assessment can be undertaken by telephone contacts and triangulated with carer reports as well as from face-to-face interviews, thus maximising available data at follow-up. Although the TUS can be used to assess engagement in a range of structured activities (e.g. structured leisure and sports activities, socialising, etc), the focus of the current study was work, education and voluntary work. Total number of hours spent in paid work over the last year was also recorded.

#### 2.3.2 Secondary outcomes

##### 2.3.2.1 Positive and Negative Syndrome Scale (PANSS; Kay et al., 1987)

The PANSS is a 30-item rating scale developed to assess symptoms associated with psychosis. Symptoms occurring over the last week were rated. PANSS total scores were used.

##### 2.3.2.2 Beck Hopelessness Scale (BHS; Beck and Steer, 1988)

The BHS is a 20-item self-report scale designed to assess the way an individual perceives the future. Items are rated using a dichotomous true/false response format. Total scores from the BHS were used.

### 2.4 Procedure

The extended follow-up was not part of the original ISREP trial protocol and thus ethical approval was sought and granted to recontact and reconsent study participants. Participants who had consented to take part in the ISREP study were contacted by letter and telephone to invite them to take part in the follow-up assessment. Following informed consent, assessments were conducted by trained research assistants who were blind to treatment allocation. Where possible, assessments were conducted using face-to-face interviews and this occurred in 75% of cases. However, the primary outcome measure could also be administered via telephone or discussions with care co-ordinators.

### 2.5 Statistical analyses

We first report frequencies for engagement in competitive employment, voluntary work, and education at 2-year follow-up for participants with affective and non-affective early psychosis and descriptive statistics for secondary outcomes. Chi-square tests are used to test for any significant differences in engagement in work, education, and voluntary work between the treatment and control group. Where the expected count was < 5 for > 20% of the cells, Yates' corrections were employed.

Analysis of Covariance (ANCOVA) models were used to test the significance of differences on secondary outcome variables between the treatment and control groups. For each ANCOVA, outcome at the 2 year follow-up was used as the dependent variable; allocation to treatment, centre, and diagnosis were used as fixed factors; and three key variables assumed to be associated with outcome and predictive of drop out were used as covariates (baseline scores on the dependent variable; baseline schizotypal symptoms score; and length of unemployment). Non-significant interactions were removed before final testing for main effects.

## 3 Results

Frequency of engagement in work, education, and voluntary work at 2 years is shown in Table 1. Descriptive statistics for other outcome variables are given in Table 2. These are broken down by treatment and diagnostic group.

**Table 1:** Presence of paid employment, education, and voluntary work in the year following the end of the intervention period.

		N (%) engaged in activity		p-Value
		TAU (N = 37)	CBT (N = 29)	
Paid work	Total sample	6 (16.2)	9 (31.0)	0.15
	Non-affective	0 (0.0)	5 (25.0)	0.03*
	Affective	6 (46.2)	4 (44.4)	0.94
Education	Total sample	19 (51.4)	11 (38.0)	0.28
	Non-affective	14 (58.3)	10 (50.0)	0.31
	Affective	5 (38.5)	1 (11.1)	0.35
Voluntary work	Total sample	17 (46.0)	14 (48.3)	0.55
	Non-affective	12 (50.0)	11 (55.0)	0.11
	Affective	5 (38.5)	3 (33.3)	0.84

**Table 2:** Descriptive statistics – mean (SD) – by treatment and diagnosis.

		Total sample		Non-affective		Affective	
		TAU	SRT + TAU	TAU	SRT + TAU	TAU	SRT + TAU
PANSS total	T1	56.0 (10.3)	57.6 (11.6)	58.1 (9.4)	57.5 (10.8)	52.1 (11.0)	58.0 (13.4)
	T2	50.4 (10.1)	50.5 (9.2)	53.2 (8.3)	50.3 (8.2)	44.5 (11.3)	50.7 (11.3)
	T3	46.7 (12.8)	49.0 (12.2)	49.3 (11.4)	47.1 (11.4)	41.4 (14.5)	52.6 (13.8)
Beck Hopelessness Scale	T1	8.7 (5.8)	8.9 (5.8)	8.0 (5.5)	8.3 (5.5)	10.2 (6.4)	10.2 (6.3)
	T2	7.9 (5.8)	6.4 (4.7)	8.2 (5.9)	4.9 (2.3)	7.3 (5.9)	9.3 (6.6)
	T3	6.1 (6.0)	6.0 (5.3)	6.0 (6.1)	4.7 (4.8)	6.4 (6.2)	9.6 (5.5)

Note. T1 = baseline assessment, T2 = post-treatment (9 months), T3 = 2-year follow-up assessment. *p < .05.

### 3.1 Engagement in work, education and voluntary work

In the combined sample of individuals with affective and non-affective psychosis, more individuals in the SRT + TAU group had engaged in paid work over the 15 months since the end of the intervention period compared to the TAU alone group (31.0% vs. 16%). However, there were no significant differences between the SRT + TAU and TAU alone groups in terms of engagement in work, education or voluntary work. The 9 individuals from the SRT + TAU group who had engaged in work reported having done so for an average of 305.39 h over the follow-up period (SD = 334.40 h, range = 8.0–940.5 h). Data on hours spent in paid work was available for 4 of the 6 individuals from the TAU group (mean hours = 265.13, SD = 105.60, range = 108.0–332.5).

In the non-affective psychosis TAU group, 0 out of 24 participants had engaged in paid employment in the year following the end of the intervention period, compared with 5 out of 20 (25%) participants in the non-affective psychosis SRT + TAU group. This difference was found to be significant using a chi-square test with Yates' correction (expected count < 5 in > 20% cells), χ^2^(1, 44) = 4.52, p = 0.03. The 5 individuals who had engaged in work reported having done so for an average of 162 h over the follow-up period (SD = 128.09 h, range = 35–315 h). There was no difference between the non-affective SRT + TAU and TAU groups in terms of engagement in education or voluntary work.

There were no significant differences between the SRT + TAU and TAU alone groups for those with affective psychosis in terms of frequency of engagement in paid work (44.4% vs. 46.2%). The 4 individuals with affective psychosis from the SRT + TAU group who had engaged in paid work reported having done so for an average of 484.63 h (SD = 446.34 h, range = 8.0–940.5 h). Data on hours spent in paid work over the follow-up period was available for 4 of the 6 individuals with affective psychosis from the TAU group (mean = 265.13 h, SD = 105.60 h, range = 108.0–332.5 h). There was no difference between the affective SRT + TAU and TAU groups in terms of engagement in education or voluntary work.

### 3.2 Secondary outcomes

Both the TAU and SRT + TAU groups showed a gradual reduction in symptoms over the study period. At 2-year follow-up there was a strong trend suggesting an allocation by diagnosis interaction for hopelessness, with the non-affective psychosis treatment group scoring lower on the BHS than individuals in the non-affective psychosis control group (F(1,32) = 3.39, p = 0.08). However, ANCOVAs revealed no main effects of treatment on symptoms in the total sample or in the affective or non-affective psychosis subgroups.

## 4 Discussion

### 4.1 Summary of findings

The follow up data for the ISREP trial provide supportive evidence for longer term gains in the use of SRT in young people with early non-affective psychosis. Fifteen months after the end of the intervention, 25% of participants in the SRT + TAU group had engaged in paid work compared to none of the TAU group. In addition to this there was no worsening of symptoms, despite increased engagement in activity; and there was also a suggestion that improvements in hope were maintained. Engagement in other types of activity (work and voluntary work) was equivalent for the SRT + TAU and TAU non-affective psychosis groups with over 50% of both groups engaging in education and voluntary work. This is positive and suggests that some improvement in functioning may take place naturally over time. However, in order to meet longer-term goals in relation to engagement in paid work, targeted intervention is likely to be necessary.

As with the post-intervention data for ISREP reported by Fowler et al. (2009b), the positive effects of SRT seem to be specific to individuals with non-affective psychosis, with no superiority of treatment being shown for the affective psychosis sub-group. Indeed, individuals with non-affective psychosis demonstrated relatively good outcomes with over 40% engaging in education and voluntary work, irrespective of whether or not they received treatment. This replicates literature highlighting better outcomes in individuals with bipolar disorder as compared to individuals with schizophrenia, possibly due to a return to good functioning between episodes (Martinez-Aran et al., 2007). Individuals with affective psychosis may also have different barriers to functional recovery which require a different intervention. However, it must be remembered that the affective psychosis subgroup in this study was small (n = 22; 13 = TAU, 9 = SRT + TAU) and this impacts upon our ability to draw definitive conclusions.

### 4.2 Adding to the evidence-base for social recovery interventions

This study adds to the growing evidence base for the use of psychological interventions to target social and functional disability following psychosis (Kern et al., 2009). Other interventions include supported employment, Social Skills Training, and Cognitive Remediation. However, whereas other interventions tend to focus on individual barriers to recovery (e.g. cognitive deficits), SRT uses an individualised formulation combined with assertive outreach techniques to understand and target a range of barriers and comorbidity. It is also appropriate for individuals who may be ambivalent about change and who demonstrate a pattern of disengagement. As such, our study includes individuals who may not currently be considered suitable for psychological therapy. In addition, SRT differs from traditional CBT for psychosis due to its wider focus on functioning and an emphasis on the use of behavioural techniques.

It is difficult to compare the results of the current study with other interventions due to the use of different outcome measures. A review of supported employment studies in individuals with first episode psychosis (Bond et al., 2015) reports an employment rate of 49% for those receiving supported employment interventions compared to 29% of individuals receiving standard early intervention service provision. Similarly, a meta-analysis of the international evidence for supported employment for people with severe mental illness suggests that individuals in receipt of supported employment interventions are more than twice as likely to find competitive work than those receiving standard care (Modini et al., 2016). Although the employment rates in the current study are not quite as high as those from some supported employment trials, it should be remembered that supported employment is generally designed for individuals who are motivated to find work. SRT may be suitable for more chronic and complex cases that may not be ready to engage with supported employment. Indeed, the rates of employment were very low in the TAU group in the current study. This suggests that without targeted intervention, such individuals are likely to remain unemployed and socially disabled. Moreover, some of the reported challenges to implementing supported employment (Craig et al., 2014), including fears around relapse from family members and mental health team staff, may be addressed by the systemic components of our SRT intervention.

### 4.3 Study limitations

Although all participants in the trial were accessing secondary mental health services and therefore were in regular contact with mental health professionals as part of TAU, there was no control condition. Future studies should aim to compare SRT to a control intervention matched in terms of frequency of contacts and other non-specific factors. It was also not possible to follow-up all participants who were initially entered into the ISREP study and thus the effect of drop-out is not known. However, we did manage to follow-up 86% of participants, which is comparable to many other RCTs (Walters et al., 2017). It would have been interesting to look at time spent in a broader range of activities, such as structured leisure and sports activities. Indeed, the TUS was specifically developed to do this. However, this would have required all participants to have engaged with a face-to-face follow-up assessment. The decision was taken to focus on a more limited assessment of functioning which could be assessed via the telephone and from informants in order to maximise follow-up rates.

### 4.4 Conclusions and future research

Overall, evidence for the use of SRT with young people with complex social recovery problems associated with non-affective psychosis is growing. This is a highly challenging group to work with who are difficult to engage and present with complex and comorbid difficulties. However, as cases with the worst prognosis it is highly important to target this group as otherwise the likelihood is of long term social disability is high. SRT shows good promise. The SUPEREDEN3 study shows definitive evidence of a gain in activity as a result of treatment at 9 months. Benefits over the longer term are suggestive from modelling of the SUPEREDEN3 study at 6 months post-intervention and from the ISREP follow-up data presented here.

Research has suggested that social disability may precede the onset of psychosis. As such, we are in the process of conducting a trial of SRT with individuals with At Risk Mental States who have social recovery problems (PRODIGY trial; Fowler et al., 2017a, Notley et al., 2015). Findings from the PRODIGY trial will suggest whether or not these gains can be replicated in individuals at an earlier stage of illness. Further research is also necessary to explore whether SRT could be effective for individuals at a later stage of illness, outside of Early Intervention Services.
